# Improved Differential Cryptanalysis of the Ultra-Lightweight Block Cipher PICO

**DOI:** 10.3390/e28091006

**Published:** 2026-09-08

**Authors:** Yu Wang, Zhuofeng Liang, Ting Fan, Tao Zhou

**Affiliations:** 1 College of Computer Science and Engineering, Guilin University of Technology, Guilin 541006, China; 2007002@glut.edu.cn (Y.W.); 2120241312@glut.edu.cn (Z.L.); 2120251320@glut.edu.cn (T.Z.); 2 Guangxi Key Laboratory of Embedded Technology and Intelligent System, Guilin 541006, China

**Keywords:** differential cryptanalysis, equivalent-round-key filtering, SAT, MILP

## Abstract

PICO is an ultra-lightweight substitution–permutation network block cipher designed for resource-constrained devices such as Internet of Things terminals and edge agents. For fixed endpoints, summing the characteristic probabilities over an enumerated finite weight window gives a verifiable lower bound on the differential probability. We use a PICO-specific workflow that combines mixed-integer linear programming bounds on active substitution boxes, exact-weight Boolean satisfiability search, optional Matsui pruning, and fixed-endpoint enumeration. For the selected endpoints, enumeration over W=63,…,76 and W=66,…,79 gives finite-window lower bounds of $2^{-59.95}$ and $2^{-61.95}$ for 21 and 22 rounds, respectively. We prepend two rounds and append three rounds to the 21-round differential distinguisher. The resulting 26-round analysis is an analytical equivalent-round-key filtering-and-ranking procedure for a 108-bit tuple. The verified 21-round finite-window probability input is a factor of $2^{0.8032}\approx 1.745$ larger than the previously reported input, increasing the expected right-tuple support at fixed *S* under the analytical accounting. For the illustrative choice $S=2^{42}$, the analytical resources are $D=2^{62}$ chosen plaintexts, a normalized substitution-box filtering workload of $T=2^{100.11}$ equivalent 26-round encryptions, and $M=2^{62}$ stored plaintext–ciphertext records. This setting is not tied to a demonstrated success probability and does not establish an equal-success complexity advantage over prior work.

## 1. Introduction

With the proliferation of artificial intelligence (AI), edge agents are increasingly deployed in physical environments, where limited computation, storage, and energy budgets are typically encountered. Under such resource constraints, traditional cryptographic primitives of high complexity are rendered inapplicable for direct deployment. Consequently, lightweight block ciphers are regarded as a viable alternative for these constrained environments. PRESENT [1], GIFT [2], and SKINNY [3] are representative lightweight substitution–permutation network (SPN) block ciphers with efficient hardware and software implementations. LBlock [4] is a representative lightweight Feistel cipher, whereas LLBC [5] was designed for low-latency applications. PICO [6] is an ultra-lightweight block cipher proposed by Bansod et al. with a block length of 64 bits and a key length of 128 bits. It has a low hardware cost and can be implemented efficiently in both software and hardware.

Differential cryptanalysis [7] is recognized as a fundamental tool for block cipher security evaluation. A differential characteristic specifies one complete sequence of intermediate differences. The complete differential hull for fixed input and output differences is the set of all such characteristics, and its differential probability is the sum of their probabilities [8]. The central principle is that both the data requirement and the success probability of a key-recovery attack depend on the differential probability. The clustering effect of differential characteristics has been repeatedly demonstrated in the cryptanalysis of lightweight ciphers. For example, the analysis of SIMON and SIMECK by Leurent et al. [9] shows that accounting for this effect can noticeably change the number of rounds that can be attacked and reduce the attack complexity. However, a complete differential hull can contain prohibitively many characteristics, so practical enumeration often uses a finite weight window. The sum over a verified window is a lower bound on the differential probability for the same endpoints. Enlarging the verified window adds explicitly counted contributions and produces a larger lower bound.

Currently, the automated search for differential characteristics relies on two types of models. The first is Mixed-Integer Linear Programming (MILP). Mouha et al. [10] used it to search for the number of active substitution boxes (S-boxes). Sun et al. [11] refined the model to the bit level and obtained both a lower bound on the number of active S-boxes and best differential characteristics. The other popular model is Boolean Satisfiability (SAT) or Satisfiability Modulo Theories (SMT). Mouha and Preneel [12] used SAT to search for differential characteristics of an addition–rotation–exclusive OR (ARX) structure, where XOR denotes exclusive OR. Kölbl et al. [13] applied SAT to search for the differential and linear characteristics of the SIMON family. Furthermore, Sun et al. [14] encoded Matsui’s bounding conditions as Boolean constraints to accelerate the search. MILP and SAT models have advantages in different respects. Bounds such as the minimum number of active S-boxes are readily obtained by MILP, whereas the search for best characteristics under Differential Distribution Table (DDT) constraints becomes increasingly expensive as the number of rounds grows. The SAT model allows a precise description of differential propagation, but bounds are not derived as quickly. It requires an iterative search from low weights, and the search efficiency decreases significantly as the number of rounds increases. Taka et al. [15] proposed a divide-and-conquer SAT framework that combines MILP preprocessing with SAT to search efficiently for optimal differential and linear characteristics, and evaluated the clustering effect for several ciphers by enumerating characteristics with fixed endpoints. However, they did not extend the enumeration of characteristics contributing to PICO differentials to higher weights or use it to mount a key-recovery attack. Kim et al. [16] instead searched for differential distinguishers tailored to key-recovery attacks and exploited the clustering effect to raise the probability of the distinguisher: for PIPO-64-128, they substantially reduced the time complexity while keeping the number of attacked rounds at nine. For PIPO-64-256, they gave a 13-round differential attack, two rounds more than the previous 11-round attack.

The security of PICO has been extensively studied. In [17], a branch-and-bound search was conducted, and a 21-round differential characteristic with at least 24 active S-boxes and probability $2^{-63}$ was reported. Ma et al. [18] carried out zero-correlation linear cryptanalysis on PICO, and seven-round multidimensional zero-correlation characteristics were constructed. They mounted a key-recovery attack on 10-round PICO. Shi et al. [19] used MILP to analyze the differential and linear characteristics of PICO, reproduced the reported 21-round differential characteristic mentioned above, and reported several additional characteristics of the same probability together with linear characteristics, but did not mount a key-recovery attack. Wang et al. [20] used a SAT model to sum the probabilities of differential characteristics with the same endpoints. They reported finite-window lower bounds of $2^{-60.75}$ and $2^{-62.39}$ for the 21-round and 22-round differential probabilities, respectively, and used the 21-round result in a 26-round key-recovery analysis with data, time, and memory complexities of $2^{63}$, $2^{101.11}$, and $2^{63}$, respectively. Ref. [20] enumerated the selected 21-round fixed-endpoint differential through W≤66. We extend this verified window and examine how the additional weight layers increase the finite-window lower bound and the expected right-tuple support at fixed *S*. Here, “improved” refers to the larger verified finite-window lower bound and the increased expected right-tuple support at fixed *S* under the common analytical accounting. It does not denote an improved complete master-key recovery or an equal-success complexity advantage.

In this paper, we construct a PICO-specific MILP–SAT workflow and use it to re-evaluate PICO’s resistance to differential cryptanalysis. For PICO, MILP first provides active-S-box lower bounds that narrow the candidate range. SAT then tests exact-weight subinstances under the DDT constraints. After the input and output differences are fixed, the subinstances are partitioned by total weight and active-S-box count, and complete characteristics are blocked one by one within each retained layer. The main contributions are as follows.

1. We construct a PICO-specific MILP–SAT workflow. MILP supplies active-S-box lower bounds, while SAT tests disjoint exact-weight and active-count subinstances under the DDT constraints. With fixed endpoints, complete characteristics are blocked individually and their contributions are aggregated by weight. Our methodological contribution is the PICO-specific integration of these established components into a workflow that yields auditable characteristic searches and finite-window probability sums.
2. For selected 21- and 22-round endpoint pairs, we extend the verified enumeration windows from the intermediate cutoffs W≤66 and W≤70, respectively, to matched 14-layer windows ending at W=76 and W=79. The resulting finite-window lower bounds increase from $2^{-60.75}$ to $2^{-59.95}$ and from $2^{-62.39}$ to $2^{-61.95}$.
3. We use the larger verified 21-round finite-window lower bound as the probability input for filtering-and-ranking equivalent-round-key candidates, following Ref. [20]. We prepend two rounds and append three rounds around the 21-round differential to form the 2+21+3 procedure for a 108-bit equivalent-round-key tuple. For *S* plaintext structures, we derive the data amount $D=S\cdot 2^{20}$, the normalized S-box filtering workload $T=S\cdot 2^{58.106}$, and the plaintext–ciphertext-record storage $M=S\cdot 2^{20}$. At the illustrative setting $S=2^{42}$, these quantities are $2^{62}$, $2^{100.11}$, and $2^{62}$, respectively. This setting is not tied to a demonstrated success probability. These values do not represent a comparison with Ref. [20] at the same success probability.

The remainder of this paper is organized as follows. Section 2 describes the PICO cipher. Section 3 presents the PICO-specific MILP–SAT workflow. Section 4 introduces the differential-characteristic search and the analytical equivalent-round-key filtering-and-ranking procedure for 26-round PICO, and Section 5 concludes the paper.

## 2. The PICO Cipher

This section describes the encryption procedure and key schedule of PICO.

### 2.1. Notation

The main notation used in this paper is listed in Table 1.

### 2.2. Encryption Procedure

The block length of PICO is 64 bits, the key length is 128 bits, and the number of rounds is 32. Its 64-bit state is arranged in a 4×16 matrix, as shown in (1).

(1) $$P=\left(\begin{array}{ccccccc} p_{0,15} & p_{0,14} & p_{0,13} & \cdots & p_{0,2} & p_{0,1} & p_{0,0}\\ p_{1,15} & p_{1,14} & p_{1,13} & \cdots & p_{1,2} & p_{1,1} & p_{1,0}\\ p_{2,15} & p_{2,14} & p_{2,13} & \cdots & p_{2,2} & p_{2,1} & p_{2,0}\\ p_{3,15} & p_{3,14} & p_{3,13} & \cdots & p_{3,2} & p_{3,1} & p_{3,0} \end{array}\right).$$

We use zero-based global bit indices and define $p_{i,j}=p_{16i+j}$ for 0≤i<4 and 0≤j<16. Within each 16-bit row word, j=0 denotes the least-significant bit. Hence, row *i* is written as $p_{i,15}\left\|\cdots \right\|p_{i,0}$. The same convention is used for hexadecimal differences and key-bit positions throughout the paper.

**Figure 1:**
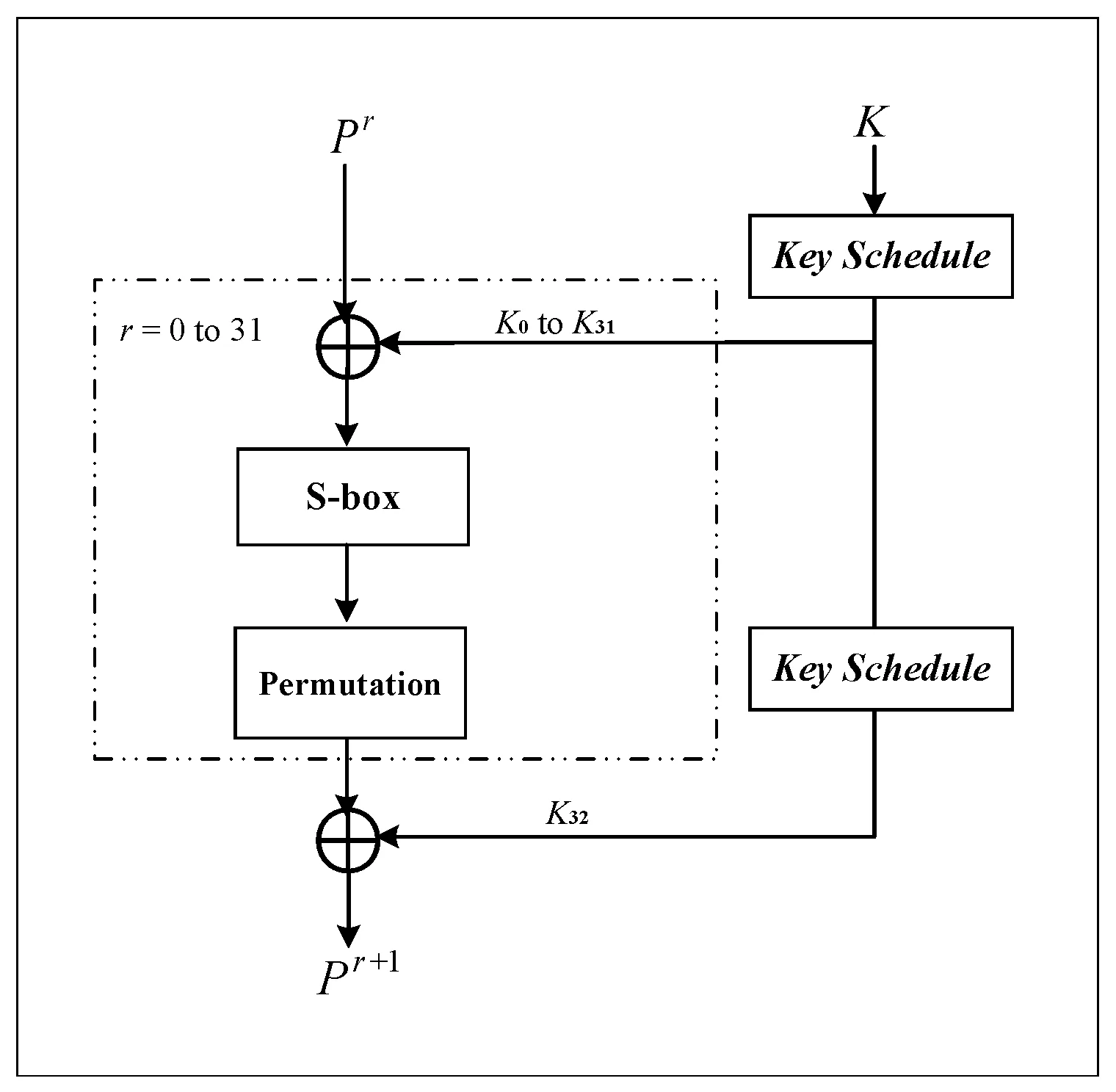
Encryption structure of PICO. S-box: substitution box.

The encryption procedure of PICO is shown in Figure 1. Starting from the plaintext *P*, for each round r=0,1,…,31, the state is XORed with the round key $K_{r}$, passed through the S-box layer, and then permuted by $P_{L}$. After the 32 rounds, the resulting state is XORed with the post-whitening key $K_{32}$ to produce the ciphertext *C*.

(1) Round Key Addition

The state matrix is XORed bitwise with the round subkey.

(2) S-box Substitution

The S-box transformation is applied to each column of the state matrix. For column *j*, the S-box input is $\left(p_{3,j}\;\|\;p_{2,j}\;\|\;p_{1,j}\;\|\;p_{0,j}\right)$ and the output is $\left(q_{3,j}\;\|\;q_{2,j}\;\|\;q_{1,j}\;\|\;q_{0,j}\right)$. The S-box lookup table is given in Table 2 (in hexadecimal).

(3) Permutation Layer

The permutation layer is a bit-level linear operation, denoted by $P_{L}$. For example, $p_{0,0}\to p_{0,10}$ means that the bit in row 0, column 0 of the state matrix is moved to row 0, column 10 of the new state matrix. The permutation layer of PICO is shown in Table 3.

### 2.3. Key Schedule

Denote the 128-bit master key by $K=k_{127}k_{126}\cdots k_{1}k_{0}$, its left 64 bits by $L_{1}=k_{127}k_{126}\cdots k_{65}k_{64}$, and its right 64 bits by $K_{0}=k_{63}k_{62}\cdots k_{1}k_{0}$, where $K_{0}$ is the round key of the first round. The remaining subkeys $K_{1}$ through $K_{32}$ are generated iteratively as follows (0≤r≤31):

(2) $$\begin{array}{cc} & L_{2}=\left(K_{r}⊕\left(L_{1}⋙3\right)\right)⊕L_{1}, \end{array}$$

(3) $$\begin{array}{cc} & K_{r+1}=\left(L_{2}⊕\left(K_{r}⋘7\right)\right)⊕r, \end{array}$$

(4) $$\begin{array}{cc} & L_{1}=L_{2}. \end{array}$$

This schedule generates 33 64-bit subkeys $K_{0},K_{1},\ldots ,K_{32}$ in total, where $K_{0}$ through $K_{31}$ are the round keys and $K_{32}$ is the whitening key applied after the final round. Like the initial state matrix, each 64-bit round subkey is arranged in a 4×16 matrix, as shown in (5).

(5) $$K_{i}=\left(\begin{array}{ccccccc} k_{0,15} & k_{0,14} & k_{0,13} & \cdots & k_{0,2} & k_{0,1} & k_{0,0}\\ k_{1,15} & k_{1,14} & k_{1,13} & \cdots & k_{1,2} & k_{1,1} & k_{1,0}\\ k_{2,15} & k_{2,14} & k_{2,13} & \cdots & k_{2,2} & k_{2,1} & k_{2,0}\\ k_{3,15} & k_{3,14} & k_{3,13} & \cdots & k_{3,2} & k_{3,1} & k_{3,0} \end{array}\right).$$

In single-key differential cryptanalysis, XOR with a round subkey does not alter differential propagation. Therefore, only the S-box layer and the bit-permutation layer are modeled in later sections.

## 3. PICO-Specific MILP–SAT Workflow for Differential Cryptanalysis

MILP optimizes an objective function subject to a set of linear constraints and can be solved by solvers such as Gurobi. SAT decides whether a given formula in Conjunctive Normal Form (CNF) admits a satisfying assignment, and modern solvers such as CaDiCaL can handle large instances efficiently. MILP efficiently computes active-S-box bounds but becomes expensive for minimum-weight characteristic searches under detailed DDT constraints. SAT models differential propagation exactly, but it must test candidate weights iteratively, and lower-weight unsatisfiable (UNSAT) instances become more costly as the round count grows. This section presents a PICO-specific workflow that combines MILP active-S-box bounds, exact-weight SAT subinstances, optional Matsui pruning, and fixed-endpoint characteristic enumeration. MILP narrows the candidate range, SAT tests whether a characteristic exists at each retained weight, and fixed-endpoint SAT enumeration supplies the weighted characteristic counts.

### 3.1. MILP Model for Active-S-Box Bounds

We describe the MILP model used to compute lower bounds on the number of active S-boxes in PICO.

**Definition** **1.** *Let an n-bit difference be $\Delta =(\Delta_{0},\Delta_{1},\ldots ,\Delta_{n-1})$. Its corresponding bit activity indicator variables $x=(x_{0},x_{1},\ldots ,x_{n-1})$ are defined as follows.*

(6) $$x_{i}=\left\{\begin{array}{cc} 0, & \Delta_{i}=0,\\ 1, & \Delta_{i}=1, \end{array}\right.\;0\leq i\leq n-1,$$

*that is, $x_{i}=1$ if and only if the i-th bit of the difference is nonzero (the bit is active).*

**Definition** **2.** *Let $A_{t}\in \left\{0,1\right\}$ indicate whether the t-th S-box is active.*

(7) $$A_{t}=\left\{\begin{array}{cc} 1, & \mathrm{the}\;t\text{-}\mathrm{th}\;S\text{-}\mathrm{box}\;\mathrm{is}\;\mathrm{active},\\ 0, & \mathrm{otherwise}. \end{array}\right.$$

#### 3.1.1. XOR Operation

For a single-bit difference relation a⊕b=c, a dummy variable *d* is introduced and the following constraints are imposed:

(8) $$\left\{\begin{array}{c} a+b+c\geq 2d,\\ d\geq a,\;d\geq b,\;d\geq c,\\ a+b+c\leq 2. \end{array}\right.$$

Here *d* is a dummy variable used to ensure that the number of ones among *a*, *b*, and *c* is even. The constraints thus model the feasible differential propagation of a⊕b=c and rule out impossible combinations of values.

#### 3.1.2. S-Box Activity Constraints

For the *t*-th S-box with *w* input bits and *v* output bits, let $x_{t,k}^{\mathrm{in}}\in \left\{0,1\right\}$ denote the activity indicator of its *k*-th input bit, where 0≤k≤w−1. Then $A_{t}=1$ if and only if these input bits are not all zero, which can be expressed as

(9) $$\left\{\begin{array}{c} A_{t}-x_{t,k}^{\mathrm{in}}\geq 0,\;k\in \left\{0,\ldots ,w-1\right\},\\ \sum_{k=0}^{w-1}x_{t,k}^{\mathrm{in}}-A_{t}\geq 0. \end{array}\right.$$

The first *w* constraints guarantee that $A_{t}=1$ whenever any $x_{t,k}^{\mathrm{in}}=1$. The last constraint forces $A_{t}=0$ when $x_{t,k}^{\mathrm{in}}=0$ for all *k*.

#### 3.1.3. The Inequality Description of the DDT

To characterize all feasible differential propagations through the S-box, we use SageMath 9.2 to represent the feasible DDT patterns as *m* inequalities.

(10) $$\sum_{j=0}^{n-1}\alpha_{i,j}x_{j}+\alpha_{i,n}\geq 0,\;i=0,1,\ldots ,m-1.$$

Here, n=w+v, $x_{0},\ldots ,x_{w-1}$ and $x_{w},\ldots ,x_{n-1}$ are the input- and output-bit activity indicators of the S-box, respectively. The coefficients $\alpha_{i,j}$ are generated by SageMath. Sun et al. used a greedy algorithm to remove redundant inequalities from the convex hull [11]. Sasaki et al. proposed a more compact inequality-reduction algorithm [21], which removes more redundant inequalities and reduces the number of constraints.

#### 3.1.4. The Objective Function

For the *R*-round model, let $x_{p}^{\left(r\right)}$ and $s_{p}^{\left(r\right)}$ (0≤r<R, 0≤p<64) denote the input- and output-difference bits, respectively, of the S-box layer in round *r*. Let $x^{\left(R\right)}$ denote the output difference after the permutation of the final round. The S-box inequalities are imposed on the four input and four output difference bits of S-box *k* in round *r* (where 0≤r<R and 0≤k<16). If π(p) is the output position of bit *p* under $P_{L}$, the inter-round linking constraint is $x_{\pi (p)}^{\left(r+1\right)}=s_{p}^{\left(r\right)}$ for every 0≤r<R and 0≤p<64. Finally, an additional constraint $\sum_{j=0}^{63}x_{j}^{\left(0\right)}\geq 1$ is added to exclude the all-zero input difference.

The objective is $\min\sum_{r=0}^{R-1}\sum_{k=0}^{15}A_{r,k}$. All constraints are written to a linear programming (LP) file, and the MILP model is solved with the Gurobi optimizer. The resulting minimum objective value is the minimum number of active S-boxes for reduced-round PICO. It should be emphasized that the minimum number of active S-boxes only reflects how many S-boxes are active along a characteristic and is not equal to the optimal characteristic weight. Converting this value into a weight bound and determining the probability of the best differential characteristic requires SAT to search for a valid characteristic.

### 3.2. SAT Model for Searching Valid Differential Characteristics

An *R*-round differential characteristic is a sequence of differences $C_{0}\to C_{1}\to \cdots \to C_{R}$, where $C_{0}$ is the plaintext difference and $C_{i}$ is the output difference in round *i*. The weight of such a characteristic is $W=-\log_{2}\mathrm{DP}$. All differential characteristics with the same input and output differences contribute to the same differential, whose probability is the sum of the probabilities of these characteristics.

#### 3.2.1. Variable Definitions

Let the target number of rounds be *R*. We introduce the following Boolean variables. For 0≤i<R and 0≤j<64, the variable $x_{j}^{\left(i\right)}$ is the input difference bit before the S-box layer of round *i*. The vector $x^{\left(R\right)}$ is the output difference after the permutation of the final round. Thus, $x^{\left(0\right)}$ and $x^{\left(R\right)}$ are the input and output differences in the *R*-round differential characteristic. The variable $s_{j}^{\left(i\right)}$ (0≤i≤R−1, 0≤j<64) is the difference bit after the S-box layer and before the permutation of round *i*, and $w_{k}^{\left(i\right)}$ (0≤i≤R−1, 0≤k<16) is the activity indicator of the *k*-th S-box in round *i*. In addition, a weight indicator $g_{k}^{\left(i\right)}$ is introduced for each S-box. The weight of an active S-box of PICO is two or three. We set $g_{k}^{\left(i\right)}=1$ if and only if that S-box has weight three, so that the weight of the S-box is $2w_{k}^{\left(i\right)}+g_{k}^{\left(i\right)}$. The S-box in column *k* takes as input the 4 bits of $x^{\left(i\right)}$ in the same column.

#### 3.2.2. S-Box Constraints

Let the S-box be an *m*-bit permutation (m=4 for PICO). Let $\alpha ,\beta \in {\left\{0,1\right\}}^{m}$ denote the input and output differences in an S-box, and let *w* and *g* be its activity and weight indicators. The DDT of PICO has values in {0,2,4,16}, including 159 entries with a value of 0, 72 entries with a value of two, 24 entries with a value of four, and one entry with a value of 16. The value 16 corresponds to weight 0 (w=0,g=0), the value four to weight two (w=1,g=0), and the value two to weight three (w=1,g=1), while the value 0 denotes an impossible propagation. Let A={(16,0,0),(4,1,0),(2,1,1)} denote the set of valid triples. Then all assignments for which (DDT[α][β],w,g)∉A form the set of invalid assignments

(11) $$F=\left\{(\alpha ,\beta ,w,g)|(\mathrm{DDT}[\alpha ][\beta ],w,g)\notin A\right\}.$$

For every invalid assignment (α,β,w,g)∈F, we construct the exclusion clause

(12) $$c=\overset{m-1}{\underset{j=0}{⋁}}\ell \left(\alpha_{j},\alpha_{j}^{*}\right)\vee \overset{m-1}{\underset{j=0}{⋁}}\ell \left(\beta_{j},\beta_{j}^{*}\right)\vee \ell \left(w,w^{*}\right)\vee \ell \left(g,g^{*}\right),$$

where ℓ(v,0)=v, ℓ(v,1)=¬v, and the superscript ∗ denotes the value of the corresponding component in the invalid assignment. The conjunction of all exclusion clauses describes the possible differential propagation modes of the S-box and their weights, and the Espresso algorithm is used to simplify the clause set.

#### 3.2.3. Permutation Constraints

The permutation layer of PICO is the bit permutation $P_{L}$ (see Table 3). Let π(p) denote the output position of input bit *p* under $P_{L}$. For 0≤i<R and 0≤p<64, the differential propagation is $x_{\pi (p)}^{\left(i+1\right)}=s_{p}^{\left(i\right)}$, which is encoded by the equivalence clauses $\left(x_{\pi (p)}^{\left(i+1\right)}\vee \neg s_{p}^{\left(i\right)})\wedge (\neg x_{\pi (p)}^{\left(i+1\right)}\vee s_{p}^{\left(i\right)}\right)$.

#### 3.2.4. Weight and Cardinality Constraints

The total weight of an *R*-round differential characteristic is $\Omega =\sum_{t=0}^{16R-1}\left(2w_{t}+g_{t}\right)$, where *t* ranges over all 16R S-boxes. The sequential encoding [22] converts a cardinality bound of the form $\sum_{t}z_{t}\leq c$ into CNF. Taking the activity indicators $w_{0},\ldots ,w_{16R-1}$ as an example, let 1≤c<16R. The auxiliary variable $u_{i,j}$ (0≤i≤16R−2, 0≤j≤c−1) indicates that at least j+1 of the first i+1 indicators are set to one. When the cumulative number exceeds *c*, the clause set is not satisfied. The upper bound constraint $\sum_{t}w_{t}\leq c$ is encoded as CNF. The corresponding clause sets are as follows.

(13) $$\left\{\begin{array}{c} \left(\neg w_{0}\vee u_{0,0}\right),\;\left(\neg u_{0,j}\right)\;\left(1\leq j\leq c-1\right);\\ \left(\neg w_{i}\vee u_{i,0}\right),\;\left(\neg u_{i-1,0}\vee u_{i,0}\right)\;\left(1\leq i\leq 16R-2\right);\\ \left(\neg w_{i}\vee \neg u_{i-1,j-1}\vee u_{i,j}\right)\;\left(1\leq i\leq 16R-2,\;1\leq j\leq c-1\right);\\ \left(\neg u_{i-1,j}\vee u_{i,j}\right)\;\left(1\leq i\leq 16R-2,\;1\leq j\leq c-1\right);\\ \left(\neg w_{i}\vee \neg u_{i-1,c-1}\right)\;\left(1\leq i\leq 16R-2\right);\\ (\neg w_{16R-1}\vee \neg u_{16R-2,c-1}). \end{array}\right.$$

In the released implementation, the sequential counter includes reverse clauses so that its auxiliary variables are exact prefix-sum witnesses. An equality $\sum_{t=0}^{n-1}z_{t}=q$ is imposed by combining the at-most-*q* counter with an at-least-*q* condition derived from the final counter. The boundary cases q=0 and q=n are handled by unit clauses. Exact-cardinality constraints are applied separately to the activity indicators $w_{t}$ and the weight indicators $g_{t}$, and the search and enumeration are further partitioned by the total number of active S-boxes. For a candidate weight *W*, since the weight of each active S-box is two or three, the total number *A* of active S-boxes must satisfy ⌈W/3⌉≤A≤⌊W/2⌋. For each integer *A* in this interval, we fix $\sum_{t}w_{t}=A$ and $\sum_{t}g_{t}=W-2A$ in the corresponding subinstance with the sequential encoding. Each subinstance is assigned total weight *W*. All subinstances over feasible *A* are pairwise disjoint and together form the entire search space at weight *W*. The active S-box count needed for subsequent Matsui pruning is also derived from *A*.

#### 3.2.5. Matsui Pruning

Let b(t) denote a valid MILP-derived lower bound on the number of active S-boxes in any nonzero *t*-round PICO differential characteristic, with b(0)=0. In a given (W,A) subinstance, the total number of active S-boxes is fixed exactly to *A*. To accelerate the search, Matsui pruning can be applied to every consecutive round interval. The portions on the two sides of an interval $\left[r_{1},r_{2}\right]$ require at least $b(r_{1})$ and $b(R-1-r_{2})$ active S-boxes. The number of active S-boxes $\sigma_{r_{1},r_{2}}=\sum_{i=r_{1}}^{r_{2}}\sum_{k}w_{k}^{\left(i\right)}$ satisfies

(14) $$\sigma_{r_{1},r_{2}}\leq A-b\left(r_{1}\right)-b\left(R-1-r_{2}\right).$$

For every satisfying assignment, a consecutive interval is itself a valid nonzero differential subtrail: the PICO S-box is a permutation, so a nonzero input difference cannot map to a zero output difference, and the bit permutation preserves nonzeroness. Consequently, the nonzero-input, DDT, and permutation constraints, together with the definition of $b(r_{2}-r_{1}+1)$, imply $\sigma_{r_{1},r_{2}}\geq b\left(r_{2}-r_{1}+1\right)$ as a semantic property; this relation is not duplicated as a separate Matsui pruning clause. Because the total active count is fixed to *A*, the lower bounds on the two outside portions yield the displayed upper bound. When this bound is nontrivial, the released function append_matsui_constraint() adds it as an at-most constraint on the interval activity indicators, reusing the sequential counter [14].

#### 3.2.6. Nonzero Constraint

The all-zero solution is excluded by ${⋁}_{j=0}^{63}x_{j}^{\left(0\right)}$.

#### 3.2.7. Complete Model

Combining the above constraints, the SAT subinstance for an *R*-round differential characteristic of weight *W* with exactly *A* active S-boxes is

(15) $$F_{R,W,A}=F_{\mathrm{Sbox}}\wedge F_{\mathrm{Perm}}\wedge F_{\mathrm{Weight}}\wedge F_{\mathrm{NonZero}}.$$

The weight term $F_{\mathrm{Weight}}$ fixes $\sum_{t}w_{t}=A$ and $\sum_{t}g_{t}=W-2A$ (the total weight is Ω=W). Optional Matsui pruning constraints may be conjoined with the model to accelerate solving. A satisfying assignment returned by the solver corresponds to an *R*-round differential characteristic of weight *W*. An unsatisfiable result indicates that this subinstance has no solution.

To enumerate the characteristics contributing to a differential, the input and output differences $x^{\left(0\right)}=\Delta_{\mathrm{in}}$ and $x^{\left(R\right)}=\Delta_{\mathrm{out}}$ are additionally fixed in the above subinstance. For each weight *W*, every feasible value of *A* defines a separate CaDiCaL subinstance. A weight layer is called complete only when every relevant subinstance terminates with UNSAT after all decoded characteristics have been blocked. Enumeration is capped at 100,000 characteristics per active-count subinstance; a capped subinstance does not support a completeness claim for that layer. The released driver run_full_enumeration.py invokes pico_fixed_endpoint_closure_csv.py, which imports the CNF construction in Pico-diff-paths-sat4.py together with the exact-weight partitioning in pico_weight_search.py and calls CaDiCaL. This is the implementation chain used to produce the layer counts reported in Section 4.2; the driver rejects a layer if any active-count subinstance is truncated or lacks the terminal UNSAT result. The scripts and retained records are provided in the Supplementary Materials. The recorded software versions are Gurobi Optimizer 13.0.1, CaDiCaL 3.0.0, SageMath 9.2, and Espresso through Logic Friday 1.1.4. No custom search-tuning parameters are set for the primary MILP optimization, and the SAT wrappers invoke CaDiCaL with the CNF filename and no custom solver flags. A fresh end-to-end run completed all 28 reported layers: 14 layers for 21 rounds (W=63,…,76) and 14 layers for 22 rounds (W=66,…,79). Every layer passed the completeness checks described above. In particular, the 22-round counts at W=77,78,79 were obtained by direct enumeration rather than extrapolation.

### 3.3. PICO-Specific MILP–SAT Workflow

When searching for a minimum-weight differential characteristic with a SAT model alone, lower-weight UNSAT subinstances must be resolved before minimality can be established. Each lower-weight instance must be shown unsatisfiable before the minimum weight can be established, and proving these UNSAT results becomes more costly as the number of rounds grows. In this workflow, MILP supplies the active-S-box lower bound used to initialize the exact-weight SAT search. The minimum number of active S-boxes is not necessarily equal to the minimum characteristic weight. Each active S-box of PICO has weight two or three, so b(R) active S-boxes give a weight lower bound of 2b(R), whereas the optimal weight may be larger. Therefore, MILP first computes b(R), the minimum number of active S-boxes. Then, the SAT model starts from W=2b(R). For each candidate weight *W*, it considers the feasible values of the total number *A* of active S-boxes and tests whether the corresponding subinstance has a solution. A satisfiable subinstance at weight *W* yields a feasible characteristic of probability $2^{-W}$. It establishes the minimum weight only if every subinstance at every smaller candidate weight has terminated with UNSAT. If all subinstances are unsatisfiable at that *W*, then *W* is increased by one and the test continues. The minimum active S-box counts b(t) for the other round counts (1≤t≤R−1) can also be used to construct Matsui pruning bounds that accelerate the search.

The detailed procedure of the PICO-specific workflow is given in Algorithm 1. Line 1 uses MILP to compute the minimum active-S-box count, and Line 2 converts it into a weight lower bound. For each candidate weight *W*, all feasible active-count values *A* are tested. A decoded assignment yields a feasible differential characteristic. The returned weight is minimal only when all subinstances at smaller candidate weights have been completely tested and found UNSAT; otherwise, it is reported only as the first retained feasible weight. The characteristic can then supply endpoints for the finite-window enumeration below.
**Algorithm 1** MILP–SAT search for a minimum-weight differential characteristic**Input:** Number of rounds *R*; table lb[1…R−1] of minimum active S-box counts for shorter rounds**Output:** First feasible weight $W^{*}$ and characteristic *C*  1: b←MILP_LB(R)▹ minimum active S-box count from MILP  2: W←2b▹ weight lower bound: each active S-box has weight ≥2  3: **while true do**  4:       **for** A←⌈W/3⌉ **to** ⌊W/2⌋ **do**  5:             F←BuildSAT(R,W,A,lb)▹ fix ∑w=A, ∑g=W−2A, optional Matsui(lb)  6:             **if** SAT(F) returns a model τ **then**  7:                    **return** (W,DecodeCharacteristic(τ))▹ feasible characteristic  8:             **end if**  9:       **end for**10:       W←W+111: **end while**

Once a retained feasible differential characteristic is obtained, its input and output differences $\left(\Delta_{\mathrm{in}},\Delta_{\mathrm{out}}\right)$ determine a differential. The probability of the differential is the sum of the probabilities of all characteristics with these input and output differences,

(16) $$\begin{array}{cc} \mathrm{Pr}(\Delta_{\mathrm{in}}\to \Delta_{\mathrm{out}}) & =\sum_{C_{1},\ldots ,C_{R-1}}\mathrm{Pr}\left(C_{0}\to \cdots \to C_{R}\right)\\ & =\sum_{W\geq W_{0}}N_{W}\cdot 2^{-W}, \end{array}$$

where $N_{W}$ is the number of differential characteristics of exact weight *W*, and $W_{0}$ is the minimum weight when all lower-weight subinstances have been completely resolved as UNSAT. A retained feasible weight may instead be used as the lower endpoint of a reported window. Enumeration proceeds level by level from low to high weight. The detailed procedure for enumerating the characteristics contributing to the differential is given in Algorithm 2. For each weight *W*, a SAT model with fixed input and output differences and fixed total weight is built and solved repeatedly. Each satisfying assignment τ is first decoded into a differential characteristic *C*. An *R*-round differential characteristic is uniquely determined by all of its round-boundary differences $\left\{x_{j}^{\left(i\right)}|0\leq i\leq R,\;0\leq j<64\right\}$ (the intermediate S-box output differences and the activity and weight indicators are all derived from these via the permutation and S-box relations). We take the characteristic-variable set $V_{C}=\left\{x_{j}^{\left(i\right)}\right\}$ and add to the model the blocking clause to exclude that characteristic.

(17) $$\underset{v\in V_{C},\;\tau \left(v\right)=0}{⋁}v\;\vee \underset{v\in V_{C},\;\tau \left(v\right)=1}{⋁}\neg v$$

The auxiliary variables of the sequential encoding are not in $V_{C}$ and do not take part in the blocking. The same differential characteristic is not counted repeatedly merely because the auxiliary variables take different values. If every relevant active-count subinstance terminates with UNSAT without hitting the cap, repeated solving yields the complete count $N_{W}$ for that retained layer; otherwise the recorded count is explicitly incomplete. The contribution $N_{W}\cdot 2^{-W}$ from each verified layer is then accumulated. As the weight grows, the contribution of a single characteristic decays as $2^{-W}$, and the accumulating blocking clauses slow the solver down, so the enumeration is truncated at an upper bound $W_{\max}$. The value obtained over $\left[W_{\min},W_{\max}\right]$ is a finite-window lower bound on the complete differential probability for the same endpoints. Additional valid characteristics outside the retained window can only increase the complete probability. The verified finite-window lower bound is used in the analytical outer-round filtering-and-ranking procedure below.

In the hybrid search model, the table b(t) of shorter-round minimum active S-box counts computed by MILP serves two purposes. First, b(R) provides the starting weight lower bound 2b(R) for Algorithm 1, reducing the number of low-weight unsatisfiable instances that must be resolved. A minimum-weight conclusion additionally requires complete UNSAT results for every lower candidate weight. Second, the shorter-round values b(t) can serve as Matsui pruning bounds in Algorithm 2, accelerating the solution of high-weight subinstances during fixed-endpoint characteristic enumeration. By enumerating subinstances at exact weights, the model counts more valid characteristics under fixed input and output differences and extends the enumeration to higher weights. This enlarges the verified finite-window lower bound for the fixed endpoints.
**Algorithm 2** The SAT-based enumeration of differential characteristics contributing to a differential**Input:** Number of rounds *R*; endpoints $\left(\Delta_{\mathrm{in}},\Delta_{\mathrm{out}}\right)$; weight window $\left[W_{\min},W_{\max}\right]$; minimum active S-box counts lb**Output:** Finite-window lower bound $P_{\left[W_{\min},W_{\max}\right]}$ for the fixed endpoints  1: P←0  2: **for** $W\leftarrow W_{\min}$ **to** $W_{\max}$ **do**  3:       $N_{W}\leftarrow 0$  4:       **for** A←⌈W/3⌉ **to** ⌊W/2⌋ **do**  5:             $F\leftarrow \mathrm{BuildSAT}(R,\Delta_{\mathrm{in}},\Delta_{\mathrm{out}},W,A,lb)$▹ fix endpoints, ∑w=A, ∑g=W−2A; optional Matsui(lb)  6:             **while** SAT(F) returns a model τ **do**  7:                    C←DecodeCharacteristic(τ); $N_{W}\leftarrow N_{W}+1$  8:                    add to F a blocking clause on the characteristic variables of *C*  9:             **end while**10:       **end for**11:       $P\leftarrow P+N_{W}\cdot 2^{-W}$▹$N_{W}$: number of weight-*W* characteristics12: **end for**13: **return** *P*

## 4. Finite-Window Differential Analysis and Outer-Round Filtering for 26-Round PICO

This section applies the PICO-specific workflow presented in Section 3. We obtain finite-window lower bounds for the selected 21-round and 22-round endpoint pairs. Finally, we prepend two rounds and append three rounds to the 21-round differential distinguisher to form a 26-round analytical equivalent-round-key filtering-and-ranking procedure for a 108-bit tuple.

### 4.1. Reported Differential-Characteristic Weights

**Figure 2:**
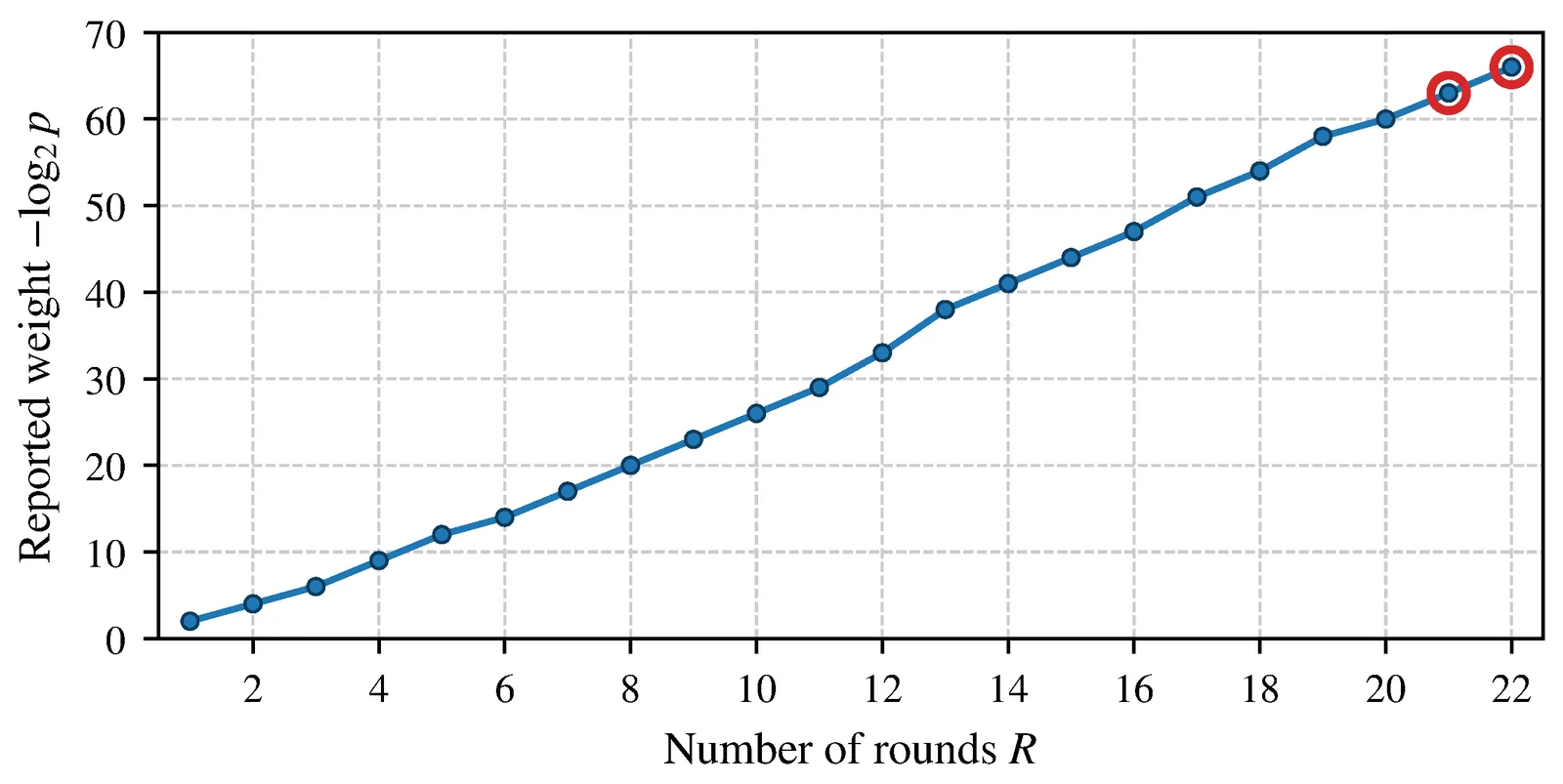
Reported differential-characteristic weight $-\log_{2}p$ versus the number of rounds. Red circles mark the 21- and 22-round values, 63 and 66.

We give the retained search results for PICO. For 21 rounds, MILP gives a minimum of 24 active S-boxes and hence a weight lower bound of 48. Starting at weight 48, SAT finds a feasible 21-round characteristic of weight 63, with 24 active S-boxes and probability $2^{-63}$. For 22 rounds, MILP gives an active-S-box lower bound of 24; SAT rules out A=24 and finds a feasible characteristic at A=25, thereby establishing a minimum active-S-box count of 25 in the retained search. The retained feasible starting weight for the 22-round characteristic search is 66. A characteristic weight is called minimum only when every lower candidate-weight subinstance has been completely resolved as UNSAT. The reported characteristic weights for PICO over 1–22 rounds are listed in Table 4. Figure 2 shows how these retained weights change with the number of rounds. The reported weight generally rises with the number of rounds. Its average increase is about 3 units per round, although adjacent-round increments fluctuate. The reported weights for 1–3 rounds are two, four, and six, respectively. From 12 rounds onward, the adjacent-round increment is 3–5 weight units, except from 19 to 20 rounds, where it is two. Correspondingly, the probability of the retained characteristic decreases as the round count grows. The 21- and 22-round retained weights marked by red circles are 63 and 66, respectively. These are adopted as enumeration starting points for the corresponding differentials below.

### 4.2. The 21-Round and 22-Round Fixed-Endpoint Differentials

The model in Section 3 uses the MILP weight lower bound to narrow the SAT search range. In the retained records, SAT yields feasible 21-round characteristics at weight 63. The endpoint selection is heuristic. We rank the retained weight-63 endpoint groups by the total Hamming weight of their input and output differences. Four retained endpoint groups attain the minimum total endpoint Hamming weight of four, with two active bits at each endpoint. Among these tied sparse groups, we fix $\Delta_{\mathrm{in}}=2000000020000000$ and $\Delta_{\mathrm{out}}=0000000804000000$ as the representative used for the subsequent finite-window and outer-round calculations. The retained endpoint-group record also lists the other tied pairs. Having few active bits at the endpoints helps limit the cost of adding rounds before and after the differential distinguisher in the outer-round filtering-and-ranking procedure. We fix this endpoint pair and enumerate the retained window W=63,…,76 according to Algorithm 2. The result is a verified finite-window contribution for the 21-round differential. Table 5 gives the retained count at each weight. The fourteen enumerated layers contribute

(18) $$\begin{array}{cc} \mathrm{Pr}(\Delta_{\mathrm{in}}\to \Delta_{\mathrm{out}}) & \geq \sum_{W=63}^{76}N_{W}\cdot 2^{-W}\\ & =\frac{68000}{2^{76}}\approx 2^{-59.9468}. \end{array}$$

Ref. [20] reported $2^{-60.75}$ for the same endpoint pair over W≤66. Extending the verified window through W=76 gives $68000/2^{76}\approx 2^{-59.9468}$. This is the verified finite-window contribution for the selected fixed endpoints.

For 22 rounds, we use $\Delta_{\mathrm{in}}=0200000002000000$ and $\Delta_{\mathrm{out}}=0000000804000000$; the output difference is shared with the 21-round pair. A retained feasible characteristic has weight 66. We enumerate the 14-layer finite window W=66,…,79. Table 6 reports the individual layer counts, whose probability contributions sum to

(19) $$\begin{array}{cc} \mathrm{Pr}(\Delta_{\mathrm{in}}\to \Delta_{\mathrm{out}}) & \geq \sum_{W=66}^{79}N_{W}\cdot 2^{-W}\\ & =\frac{136000}{2^{79}}\approx 2^{-61.9468}. \end{array}$$

**Figure 3:**
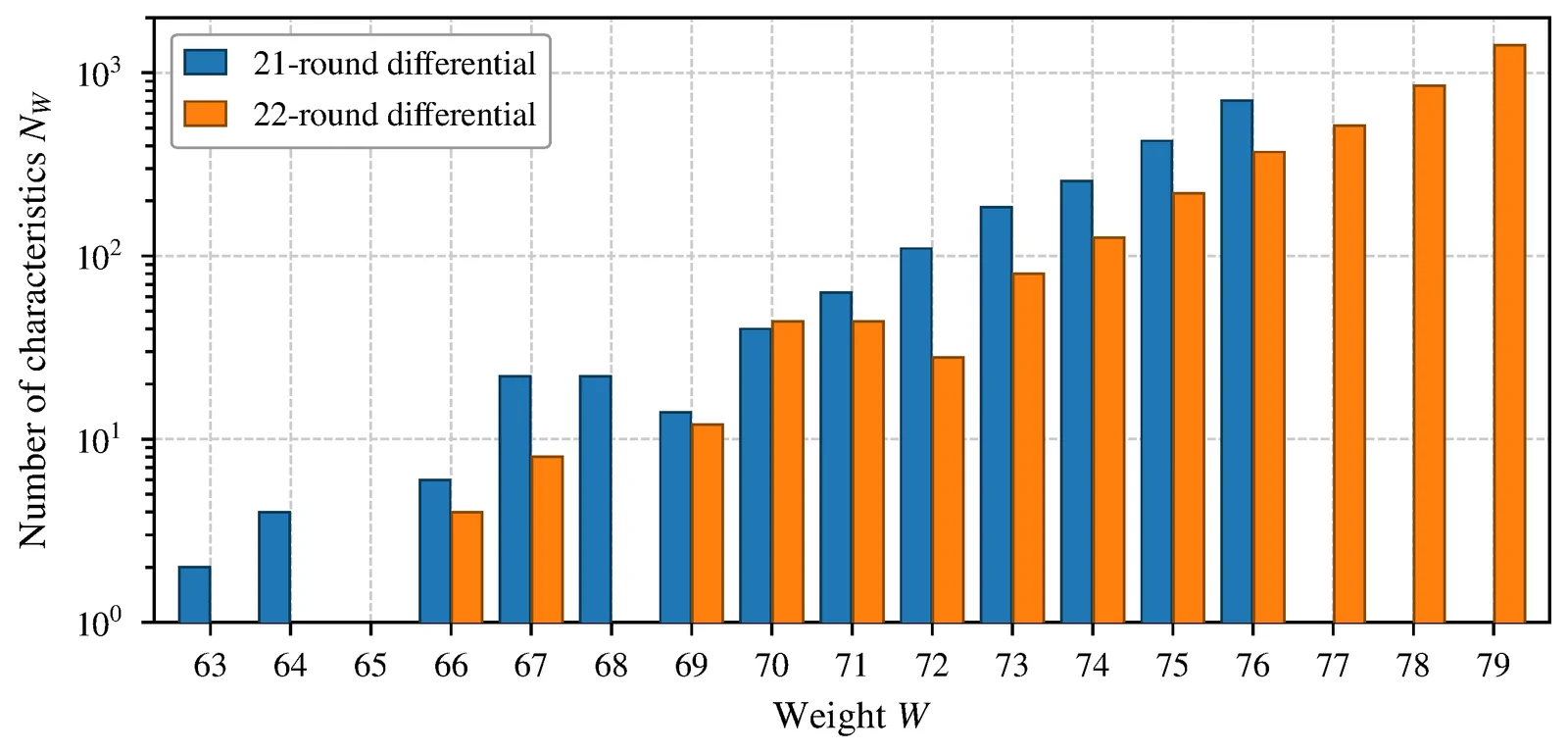
Numbers of 21-round (blue, W=63,…,76) and 22-round (orange, W=66,…,79) differential characteristics at each weight *W* for the respective fixed input–output difference pairs.

At the intermediate cutoff W≤70, the finite-window lower bound is $2^{-62.39}$; extending the verified window through W=79 gives $136000/2^{79}\approx 2^{-61.9468}$. The added layers W=77,78,79 were directly enumerated and contain 514, 850, and 1412 characteristics, respectively. In Figure 3, the counts of differential characteristics per weight are compared for the two fixed input and output differences. Within the recorded windows, $N_{W}$ rises monotonically from W=70 for 21 rounds and from W=73 for 22 rounds. For 21 rounds, it grows from two at W=63 to 706 at W=76; for 22 rounds, it grows from 80 at W=73 to 1412 at W=79. The retained 22-round starting weight is 66, three units higher than for 21 rounds. Although the probability of each characteristic decreases as $2^{-W}$, the growing number of characteristics makes the cumulative contribution of the higher-weight layers non-negligible and partially offsets this decay. Up to W=76, a total of 1856 21-round differential characteristics are enumerated. Up to W=79, a total of 3712 22-round differential characteristics are enumerated. Only 12 of the retained 21-round characteristics satisfy W≤66, while layers W = 67–76 contribute 42.78% of the finite sum through W=76. For 22 rounds, layers W = 71–79 contribute 26.21% of the finite sum through W=79. The directly enumerated layers W = 77, 78, and 79 contribute 1.5118%, 1.2500%, and 1.0382%, respectively, or 3.8000% in total. These percentages quantify the weighted additions within the two verified windows.

Let $N_{R}\left(W\right)$ denote the number of recorded *R*-round characteristics of weight *W*. Completed per-layer enumeration yields the observed equality $N_{22}\left(W\right)=2N_{21}\left(W-3\right)$ for every W=66,…,79. We use this observed equality only as a numerical cross-check, not as a proved structural identity. No 22-round count is generated from the 21-round table. A supplementary bounded short-prefix audit explains the first pair of layers: the weight-63 and weight-66 characteristics share two identical 19-round suffixes, each preceded by one two-round prefix of weight five and two three-round prefixes of weight eight, respectively. Short-prefix multiplicities vary across other interfaces, however, and this local check does not establish a global bijection for the characteristic sets within the stated windows. The probability calculation and per-layer completion checks therefore use the independently enumerated counts, not an assumed structural identity.

**Figure 4:**
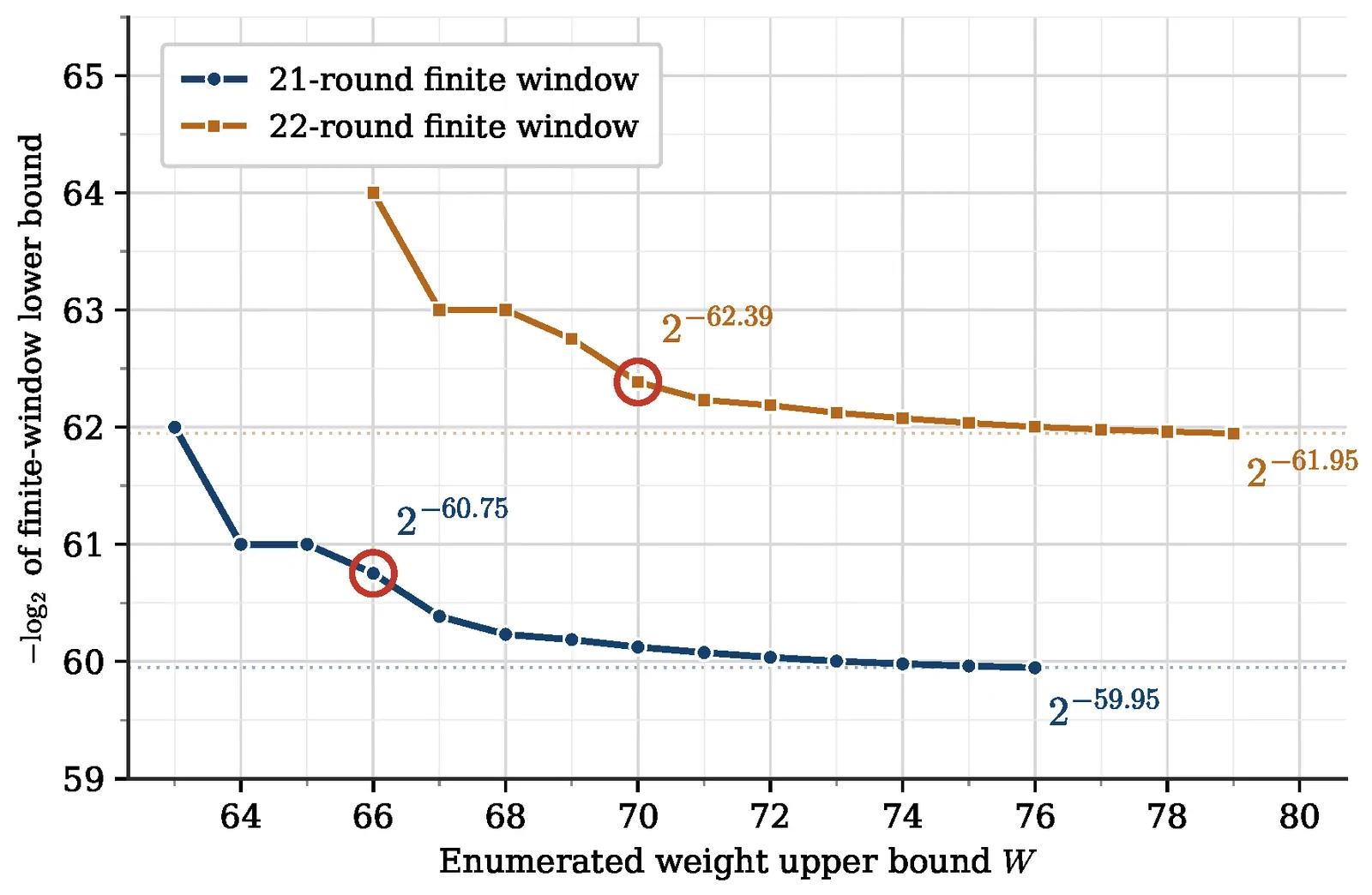
Finite-window lower bounds for the selected 21-round and 22-round endpoint pairs as functions of the upper endpoint *W*. The curves end at W=76 and W=79, respectively, so both contain 14 enumerated layers.

Table 7 compares the two finite-window lower bounds at equal relative depths from their respective starting weights. The five rows contain four, eight, 10, 12, and 14 enumerated layers. Thus, the 21-round upper endpoints W=66,70,72,74,76 are paired with the 22-round upper endpoints W=69,73,75,77,79. Figure 4 traces every completed prefix of each window. At the matched depth of 14 layers, the bounds are $2^{-59.95}$ and $2^{-61.95}$, respectively.

### 4.3. Analytical Equivalent-Round-Key Filtering and Ranking for 26-Round PICO

#### 4.3.1. Differential Distinguisher Extension

**Figure 5:**
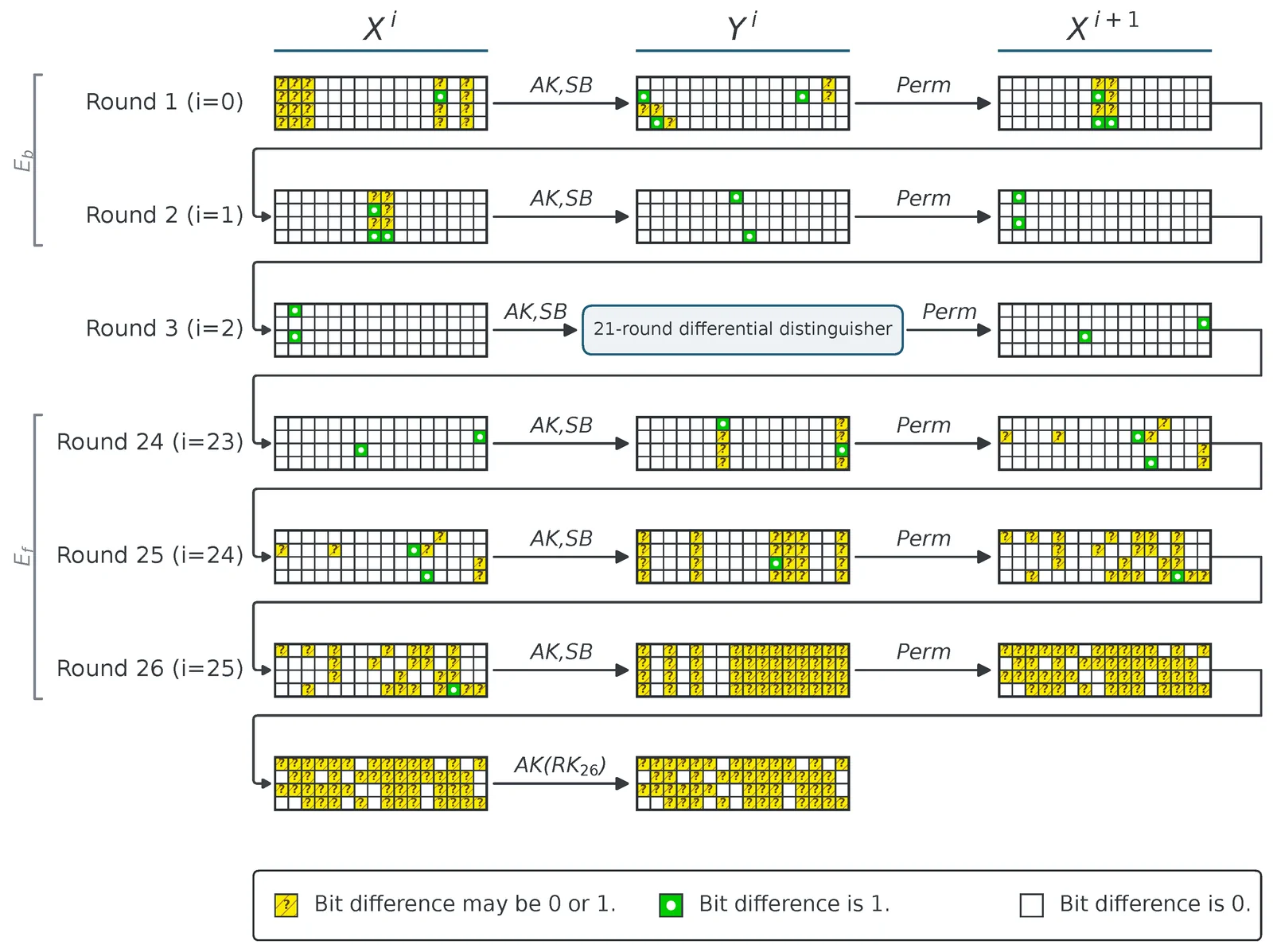
Difference propagation and per-round active bits in the analytical outer-round filtering-and-ranking procedure for 26-round PICO. AK denotes round key addition, SB denotes S-box substitution, and Perm denotes bit permutation.

The 26-round encryption is split into three parts, $E=E_{f}\circ E_{d}\circ E_{b}$. Here, $E_{b}$ covers rounds 1∼2, $E_{d}$ is the 21-round differential distinguisher over rounds 3∼23, and $E_{f}$ covers rounds 24∼26. This 21-round differential distinguisher uses the input and output differences given above. The characteristics enumerated over W=63,…,76 contribute approximately $2^{-59.9468}$ to the fixed-endpoint probability, and this verified finite-window lower bound is used in the analysis below. The difference propagation and the per-round bit conditions are shown in Figure 5. Throughout this procedure, rounds are numbered from one to 26, whereas state and key indices are zero-based. For 0≤i≤25, $X^{i}$ denotes the state difference at the input of round i+1, before the addition of $RK_{i}$, and $Y^{i}$ denotes the difference after the S-box layer of the same round and before the permutation. Hence, $X^{i+1}=P_{L}\left(Y^{i}\right)$. The 26-round version of PICO uses $RK_{0},\ldots ,RK_{25}$ as round keys and $RK_{26}$ as the post-whitening key. The equivalent round key $EK_{i}$ is defined by $P_{L}\left(EK_{i}\right)=RK_{i+1}$.

#### 4.3.2. Analytical Data Accounting

To construct the plaintext structures, we extend the input difference in the distinguisher backward through the two rounds of $E_{b}$ to obtain the plaintext-difference pattern. The plaintext difference $X^{0}$ involves 20 designated bit positions. The difference at bit 28 is fixed to one, whereas the differences at the other 19 positions may be either 0 or one. Fixing the remaining 44 plaintext bits to constant values yields a structure of $2^{20}$ plaintexts. Splitting the structure into two halves $G_{0}$ and $G_{1}$ (each of size $2^{19}$) according to the value of bit 28 and pairing across the two halves gives $2^{38}$ pairs. Taking *S* structures gives $S\cdot 2^{38}$ pairs in total, with data complexity $D=S\cdot 2^{20}$. The differential propagation probability over the first two rounds is $P_{b}=2^{-14}\cdot 2^{-5}=2^{-19}$. A pair is a right pair only if it follows the prescribed differential propagation through both $E_{b}$ and $E_{d}$. Using the verified W=63,…,76 contribution as the probability input, the expected number of right pairs supported by the enumerated window is

(20) $$N_{\mathrm{win}}=S\cdot 2^{38}\cdot 2^{-19}\cdot \frac{68000}{2^{76}}\approx S\cdot 2^{-40.9468}.$$

The output difference in the distinguisher is then propagated forward through the three rounds of $E_{f}$ to the ciphertext. Under this propagation, 16 ciphertext difference bits are fixed to zero. In the analytical accounting, filtering with these bits requires no key guesses and leaves an expected $N_{F}=S\cdot 2^{38}\cdot 2^{-16}=S\cdot 2^{22}$ surviving pairs.

Under the standard wrong-key randomization hypothesis and the assumption that the filtering conditions are approximately independent and uniformly distributed, the probability that a surviving pair casts a false vote for a given wrong tuple is $P_{w}\approx 2^{-67}$. The first two rounds provide 19 conditions and the last three rounds provide 48 conditions. Therefore, the average number of votes for one wrong tuple is $N_{\mathrm{wrong}}=S\cdot 2^{22}\cdot 2^{-67}=S\cdot 2^{-45}$. Following the classical signal-to-noise accounting introduced for differential key ranking by Biham and Shamir [7], the ratio between the expected right-tuple support and the average wrong-tuple support is

(21) $$S/N=\frac{N_{\mathrm{win}}}{N_{\mathrm{wrong}}}\approx \frac{S\cdot 2^{-40.9468}}{S\cdot 2^{-45}}=2^{4.0532}\approx 16.6.$$

Here, S/N is an analytical separation metric under the stated assumptions; it is not treated as an empirically measured ranking success probability.

#### 4.3.3. Equivalent-Round-Key Filtering Schedule

The analytical filtering schedule consists of 27 steps, listed in Table 8. Each step introduces one four-bit equivalent-round-key guess and one differential filtering condition. The schedule covers $EK_{25}$ (12 steps), $EK_{24}$ (six steps), $EK_{23}$ (two steps), $RK_{0}$ (five steps), and $RK_{1}$ (two steps), for a total of 108 equivalent-round-key bits. The analysis specifies a nominal target ranked list for this 108-bit equivalent-round-key tuple. Here $EK_{25}$, $EK_{24}$, and $EK_{23}$ lie in $E_{f}$ after the distinguisher, while $RK_{0}$ and $RK_{1}$ lie in $E_{b}$ before it. The order and filtering conditions in Table 8 follow the difference propagation for the selected endpoint pair. Summing the nominal S-box workloads in Table 8 gives $S\cdot 2^{66.807}$ S-box operations. One 26-round encryption comprises $26\times 16=416\approx 2^{8.700}$ S-box operations, so the normalized S-box filtering workload is $T=S\cdot 2^{58.106}$ equivalent 26-round encryptions. Here, *a* is used only as a target-list-size parameter. Setting a=14 specifies a target list containing the top $2^{-14}$ fraction of the nominal $2^{108}$ tuple space, namely $2^{94}$ tuple candidates. This target-list specification for the 108-bit equivalent-round-key tuple defines the terminal scope of the analysis. Because the guessed positions are drawn from several round-key and equivalent-key words linked by the PICO key schedule, the nominal 108-bit tuple size cannot be subtracted directly from the 128-bit master-key length to infer an independent remaining key space.

#### 4.3.4. Analytical Workload, Signal-to-Noise Ratio, and Output Scope

For *S* plaintext structures, the analytical data amount is $D=S\cdot 2^{20}$ chosen plaintexts, the normalized S-box filtering workload is $T=S\cdot 2^{58.106}$ equivalent 26-round encryptions, and the plaintext–ciphertext-record storage is $M=S\cdot 2^{20}$ records. The time conversion uses 416 S-box operations per 26-round encryption. The expression for *T* excludes ranking comparisons and candidate-list materialization; *M* counts only plaintext–ciphertext records and excludes candidate-list storage. As an analytical accounting convention, each listed bit condition is assigned its nominal pass factor, and these factors are multiplied across the schedule. The resulting expected pair counts and step costs in Table 8 are conditional workload terms rather than empirical key-ranking measurements.

For completeness, the normal-approximation model of Selçuk [23] expresses the probability that the correct tuple is retained in the top $2^{-a}$ fraction of candidates as

(22) $$P_{S}=\Phi \left(\frac{\sqrt{\mu \cdot S/N}-\Phi^{-1}\left(1-2^{-a}\right)}{\sqrt{S/N+1}}\right),$$

where $\mu =N_{\mathrm{win}}$, and Φ and $\Phi^{-1}$ are the cumulative distribution function of the standard normal distribution and its inverse. This equation records the analytical framework used in Ref. [20]. In the present work, it provides the theoretical context for S/N; the reported results are the verified finite-window probability input, the corresponding expected support, and the resource quantities *D*, *T*, and *M*.

We choose $S=2^{42}$ solely as an illustrative analytical setting, not as a parameter tied to a demonstrated ranking success probability. At this setting, $\mu =N_{\mathrm{win}}\approx 2^{1.0532}\approx 2.08$, and the analytical resource quantities are

(23) $$\left\{\begin{array}{cc} D & =S\cdot 2^{20}=2^{62},\\ T & =S\cdot 2^{58.106}=2^{100.11},\\ M & =2^{62}. \end{array}\right.$$

These values illustrate the resource expressions at the chosen *S* and do not establish an equal-success complexity advantage over Ref. [20]. The procedure stores $S\cdot 2^{20}=2^{62}$ plaintext–ciphertext records. At 64 bits for the plaintext and 64 bits for the ciphertext, the raw record content is 16 bytes per record, or $2^{66}$ bytes before counters, metadata, and implementation overhead. At each step, counters are maintained only for the currently surviving pairs, and filtering is performed incrementally by bit position; these operations do not change the stated asymptotic record-storage term.

The 27-step schedule has the same outer-round organization as Ref. [20], while the exact key quartets, their order, and the filtering bit positions are determined by the endpoint pair used here. Recomputing the step costs gives $S\cdot 2^{66.806553}$ S-box operations for Ref. [20] and $S\cdot 2^{66.806539}$ for the present table. After normalization by 26×16=416 S-box operations per 26-round encryption, both give $T/S\approx 2^{58.106}$, reported as approximately $2^{58.11}$ equivalent encryptions per structure. The verified finite-window probability input increases from $2^{-60.75}$ to $2^{-59.9468}$, a factor of $2^{0.8032}\approx 1.745$; hence, for any fixed *S*, the expected right-tuple support $N_{\mathrm{win}}$ is larger by the same factor under the common analytical accounting.

In Table 9, $P_{d}$ denotes the reported finite-window lower bound, and *D*, *T*, and *M* use the accounting defined above. The row from Ref. [20] retains that source’s values at $S=2^{43}$. The present row uses the illustrative setting $S=2^{42}$, for which no ranking success probability is established.

## 5. Conclusions

In this work, we integrated MILP-derived active-S-box bounds, exact-weight SAT subinstances, optional Matsui pruning, and fixed-endpoint blocking enumeration into a PICO-specific workflow. We used this workflow to re-evaluate PICO’s resistance to differential cryptanalysis. For selected endpoints, the verified enumeration windows were extended to matched relative depths of 14 layers, ending at W=76 for 21 rounds and W=79 for 22 rounds. The resulting finite-window lower bounds are $2^{-59.95}$ and $2^{-61.95}$, respectively. We prepended two rounds and appended three rounds to the 21-round distinguisher, yielding a 2+21+3 analytical equivalent-round-key filtering-and-ranking procedure over rounds 1–26. The procedure concerns a 108-bit equivalent-round-key tuple. At the illustrative setting $S=2^{42}$, the analytical data amount, normalized S-box filtering workload, and plaintext–ciphertext-record storage are $D=2^{62}$ chosen plaintexts, $T=2^{100.11}$ equivalent 26-round encryptions, and $M=2^{62}$ stored records, respectively. This setting is not tied to a demonstrated success probability, and these values do not establish an equal-success complexity advantage over Ref. [20]. Compared with Ref. [20], the extended 21-round finite window increases the verified probability input, and therefore the expected right-tuple support at any fixed *S* under the common analytical accounting, by a factor of $2^{0.8032}\approx 1.745$. This improvement concerns the verified finite-window lower bound and the corresponding analytical expected support, not a demonstrated complete master-key recovery. The outer-round result remains an analytical procedure terminating at the filtering-and-ranking stage for a 108-bit equivalent-round-key tuple.

## Figures and Tables

**Table 1:** Notation used in this paper.

Notation	Description
*P*	The 64-bit input plaintext
*C*	The 64-bit output ciphertext
$P^{r}$, $P^{r+1}$	The 64-bit input and output of round *r*
*r*	The round index, 0≤r≤31
*K*	The 128-bit master key
$K_{r}$	The subkey used in round *r*
$P_{L}$	The bit-permutation layer
**Δ**	A difference (bit vector)
DP	The probability of a differential characteristic
*W*	The weight of a differential characteristic, $W=-\log_{2}\mathrm{DP}$
⊕	The bitwise exclusive OR (XOR) operation
‖	The concatenation of two binary strings
⋘*n*	Left cyclic shift by *n* bits
⋙*n*	Right cyclic shift by *n* bits

Bold mathematical symbols denote bit vectors. Bold table headings and row labels are typographical labels, not vector notation.

**Table 2:** The S-box of PICO (in hexadecimal).

***x***	0	1	2	3	4	5	6	7	8	9	A	B	C	D	E	F
S[x]	1	2	4	D	6	F	B	8	A	5	E	3	9	C	7	0

S-box: substitution box. The first row lists inputs *x* and the second row lists outputs S[x]. A–F denote hexadecimal values 10–15. Bold identifies the row labels.

**Table 3:** The bit permutation of PICO.

i∖j	0	1	2	3	4	5	6	7	8	9	10	11	12	13	14	15
**0**	0,10	1,5	1,12	2,6	2,12	3,0	3,11	0,1	3,3	0,15	2,9	0,2	3,12	2,2	1,8	1,4
**1**	3,8	0,6	1,1	1,15	2,4	3,5	0,12	2,14	1,14	3,4	0,11	0,4	1,7	2,3	2,8	3,15
**2**	0,8	2,7	0,3	2,11	3,9	3,1	1,0	1,9	2,5	2,10	3,13	3,2	0,0	0,9	1,2	1,10
**3**	3,10	3,7	0,7	1,3	1,13	0,14	2,15	2,0	2,1	0,5	3,14	2,13	0,13	3,6	1,6	1,11

Bold labels identify source row *i* and column *j*. Each entry $i^{\prime },j^{\prime }$ is a destination row–column pair, not a decimal number.

**Table 4:** Reported differential-characteristic weights ($-\log_{2}p$) for PICO over 1–22 rounds.

**Rounds**	1	2	3	4	5	6	7	8	9	10	11
**−log_2_** ***p***	2	4	6	9	12	14	17	20	23	26	29
**Rounds**	12	13	14	15	16	17	18	19	20	21	22
**−log_2_** ***p***	33	38	41	44	47	51	54	58	60	63	66

Each block lists round counts in its first row and the corresponding characteristic weights in its second row. Bold identifies row labels.

**Table 5:** Number of differential characteristics contributing to the 21-round differential at each weight.

***W***	63	64	65	66	67	68	69	70	71	72	73	74	75	76
$N_{W}$	2	4	0	6	22	22	14	40	63	110	185	257	425	706

**Table 6:** Number of differential characteristics contributing to the 22-round differential at each weight.

***W***	66	67	68	69	70	71	72	73	74	75	76	77	78	79
$N_{W}$	4	8	0	12	44	44	28	80	126	220	370	514	850	1412

**Table 7:** Finite-window lower bounds for the selected 21-round and 22-round endpoint pairs at matched numbers of enumerated weight layers.

Number of Layers	21-Round Upper Bound	$P_{\left[63,W\right]}$	22-Round Upper Bound	$P_{\left[66,W\right]}$
4	W≤66	$2^{-60.75}$	W≤69	$2^{-62.75}$
8	W≤70	$2^{-60.12}$	W≤73	$2^{-62.12}$
10	W≤72	$2^{-60.04}$	W≤75	$2^{-62.04}$
12	W≤74	$2^{-59.98}$	W≤77	$2^{-61.98}$
14	W≤76	$2^{-59.95}$	W≤79	$2^{-61.95}$

**Table 8:** Complexity details of the analytical equivalent-round-key filtering-and-ranking procedure for 26-round PICO.

Step	Guessed Key Bits	Filtering Condition	Expected Remaining Pairs	Analytical S-Box Workload
1	$EK_{25}\left[0,16,32,48\right]$	$\Delta X^{25}\left[16,32,48\right]=0$	$S\cdot 2^{19}$	$2S\cdot 2^{26}$
2	$EK_{25}\left[7,23,39,55\right]$	$\Delta X^{25}\left[7,39,55\right]=0$	$S\cdot 2^{16}$	$2S\cdot 2^{27}$
3	$EK_{25}\left[14,30,46,62\right]$	$\Delta X^{25}\left[14,30,46\right]=0$	$S\cdot 2^{13}$	$2S\cdot 2^{28}$
4	$EK_{25}\left[15,31,47,63\right]$	$\Delta X^{25}\left[15,31,47\right]=0$	$S\cdot 2^{10}$	$2S\cdot 2^{29}$
5	$EK_{25}\left[2,18,34,50\right]$	$\Delta X^{25}\left[18,34\right]=0$	$S\cdot 2^{8}$	$2S\cdot 2^{30}$
6	$EK_{25}\left[8,24,40,56\right]$	$\Delta X^{25}\left[24,40\right]=0$	$S\cdot 2^{6}$	$2S\cdot 2^{32}$
7	$EK_{25}\left[9,25,41,57\right]$	$\Delta X^{25}\left[9,25\right]=0$	$S\cdot 2^{4}$	$2S\cdot 2^{34}$
8	$EK_{25}\left[11,27,43,59\right]$	$\Delta X^{25}\left[43,59\right]=0$	$S\cdot 2^{2}$	$2S\cdot 2^{36}$
9	$EK_{25}\left[12,28,44,60\right]$	$\Delta X^{25}\left[12,28\right]=0$	$S\cdot 2^{0}$	$2S\cdot 2^{38}$
10	$EK_{25}\left[4,20,36,52\right]$	$\Delta X^{25}\left[52\right]=0$	$S\cdot 2^{-1}$	$2S\cdot 2^{40}$
11	$EK_{25}\left[10,26,42,58\right]$	$\Delta X^{25}\left[42\right]=0$	$S\cdot 2^{-2}$	$2S\cdot 2^{43}$
12	$EK_{25}\left[13,29,45,61\right]$	$\Delta X^{25}\left[61\right]=1$	$S\cdot 2^{-3}$	$2S\cdot 2^{46}$
13	$EK_{24}\left[0,16,32,48\right]$	$\Delta X^{24}\left[0,32,48\right]=0$	$S\cdot 2^{-6}$	$2S\cdot 2^{49}$
14	$EK_{24}\left[4,20,36,52\right]$	$\Delta X^{24}\left[4,36,52\right]=0$	$S\cdot 2^{-9}$	$2S\cdot 2^{50}$
15	$EK_{24}\left[10,26,42,58\right]$	$\Delta X^{24}\left[10,42,58\right]=0$	$S\cdot 2^{-12}$	$2S\cdot 2^{51}$
16	$EK_{24}\left[11,27,43,59\right]$	$\Delta X^{24}\left[59\right]=1,\;\Delta X^{24}\left[11,43\right]=0$	$S\cdot 2^{-15}$	$2S\cdot 2^{52}$
17	$EK_{24}\left[12,28,44,60\right]$	$\Delta X^{24}\left[28,44,60\right]=0$	$S\cdot 2^{-18}$	$2S\cdot 2^{53}$
18	$EK_{24}\left[15,31,47,63\right]$	$\Delta X^{24}\left[15,31\right]=0$	$S\cdot 2^{-20}$	$2S\cdot 2^{54}$
19	$EK_{23}\left[6,22,38,54\right]$	$\Delta X^{23}\left[6,22,54\right]=0$	$S\cdot 2^{-23}$	$2S\cdot 2^{56}$
20	$EK_{23}\left[15,31,47,63\right]$	$\Delta X^{23}\left[15,47,63\right]=0$	$S\cdot 2^{-26}$	$2S\cdot 2^{57}$
21	$RK_{0}\left[0,16,32,48\right]$	$\Delta Y^{0}\left[16\right]=1,\;\Delta Y^{0}\left[0,48\right]=0$	$S\cdot 2^{-29}$	$2S\cdot 2^{58}$
22	$RK_{0}\left[1,17,33,49\right]$	$\Delta Y^{0}\left[49\right]=1,\;\Delta Y^{0}\left[1,17\right]=0$	$S\cdot 2^{-32}$	$2S\cdot 2^{59}$
23	$RK_{0}\left[2,18,34,50\right]$	$\Delta Y^{0}\left[2,18,34\right]=0$	$S\cdot 2^{-35}$	$2S\cdot 2^{60}$
24	$RK_{0}\left[12,28,44,60\right]$	$\Delta Y^{0}\left[12,44,60\right]=0$	$S\cdot 2^{-38}$	$2S\cdot 2^{61}$
25	$RK_{0}\left[14,30,46,62\right]$	$\Delta Y^{0}\left[46,62\right]=0$	$S\cdot 2^{-40}$	$2S\cdot 2^{62}$
26	$RK_{1}\left[8,24,40,56\right]$	$\Delta Y^{1}\left[8,24,40\right]=0$	$S\cdot 2^{-43}$	$2S\cdot 2^{64}$
27	$RK_{1}\left[7,23,39,55\right]$	$\Delta Y^{1}\left[7\right]=1,\;\Delta Y^{1}\left[39\right]=0$	$S\cdot 2^{-45}$	$2S\cdot 2^{65}$
Total				$S\cdot 2^{66.807}$

**Table 9:** Analytical resource accounting for 26-round PICO. The rows do not provide an equal-success comparison.

21-Round Distinguisher Probability $P_{d}$	Data Amount *D*	Normalized Filtering Workload *T*	Record Storage *M*	Reference
$2^{-60.75}$	$2^{63}$	$2^{101.11}$	$2^{63}$	[20]
$2^{-59.95}$	$2^{62}$	$2^{100.11}$	$2^{62}$	Ours

## Data Availability

A compact reproducibility archive accompanying this article contains the essential scripts, retained output records, concise instructions, and an integrity manifest supporting the reported calculations. Additional implementation details are available by contacting the authors at 2120241312@glut.edu.cn or 2024016@glut.edu.cn.

## Supplementary Materials

The following supporting information can be downloaded at https://www.mdpi.com/article/10.3390/e28091006/s1, A reproducibility archive containing the essential scripts, retained output records, concise instructions, and an integrity manifest supporting the reported calculations.

## Abbreviations

The following abbreviations are used in this manuscript:

AI	Artificial Intelligence
SPN	Substitution–Permutation Network
S-box	Substitution Box
ARX	Addition–Rotation–XOR
XOR	Exclusive OR
MILP	Mixed-Integer Linear Programming
LP	Linear Programming
SAT	Boolean Satisfiability
SMT	Satisfiability Modulo Theories
CNF	Conjunctive Normal Form
DDT	Differential Distribution Table
UNSAT	Unsatisfiable
AK	Round Key Addition
SB	S-Box Substitution
Perm	Bit Permutation
